# The Increased RNase Activity of IRE1α in PBMCs from Patients with Rheumatoid Arthritis

**DOI:** 10.15171/apb.2019.060

**Published:** 2019-08-01

**Authors:** Mahdieh Ahmadiany, Mahshid Alavi-Samani, Zahra Hashemi, Mohammad Amin Moosavi, Marveh Rahmati

**Affiliations:** ^1^Cancer Biology Research Center, Tehran University of Medical Sciences, Tehran, Iran.; ^2^Department of Biochemistry, Faculty of Advanced Sciences & Technology, Pharmaceutical Sciences Branch, Islamic Azad University, Tehran, Iran (IAUPS).; ^3^Department of Rheumatology, Imam Hossein Teaching Center, School of Medicine, Shahid Beheshti University of Medical Sciences, Tehran, Iran.; ^4^Department of Molecular Medicine, National Institute of Genetic Engineering and Biotechnology, Tehran P.O Box 14965/161, Iran.

**Keywords:** Endoplasmic reticulum stress, Inositol-requiring enzyme 1, IRE1-dependent decay, microRNA, Rheumatoid arthritis

## Abstract

***Purpose:*** Despite recent advances in the diagnosis and treatment of rheumatoid arthritis (RA), this inflammatory disease remains a challenge to patients and physicians. Recent evidence highlights the contribution of endoplasmic reticulum (ER) stress in the pathogenesis and treatment of RA. Herein, we study the expression of the ER stress sensor *inositol-requiring enzyme 1α (IRE1α)*, as well as XBP1 splicing and the regulated IRE1-dependent decay (RIDD), in peripheral blood mononuclear cells (PBMCs) from patients with RA compared with healthy controls.

***Methods:*** The PBMCs from blood samples of RA patients and healthy volunteers were isolated by a density gradient centrifugation method using Ficoll. The gene expression levels of *GRP78/ Bip, IRE1, XBP1s, micro-RNAs (miRNAs)* were evaluated by real-time PCR.

***Results:*** The expression of *GRP78, IRE1, and XBP1s * were increased in PBMCs of RA patients compared with healthy controls. We further show that the RIDD targets (miRNA-17, -34a, -96, and -125b) were downregulated in RA samples.

***Conclusion:*** This study can expand our knowledge on the importance of RNase activity of IRE1α in RA and may offer new potentials for developing novel diagnostic and/or therapeutic biomarkers.

## Introduction

Rheumatoid arthritis (RA) is a chronic inflammatory disease associated with swelling of synovial joints, systemic pain and progressive disability in movement.^1^ Although RA has relatively low prevalence in all over the world (eg., 0.37% in Iran),^2^ it is considered as a major global health concern that needs attention.^3^ The main aim of the current therapeutic protocols in RA is based on the use of anti-inflammatory drugs in order to prevent the progression of disease in the patients. However, these treatments are often associated with different efficacies and side effects, highlighting the need for new effective diagnostic and therapeutic strategies to control the disease.^1,4^

The endoplasmic reticulum (ER) is responsible for many essential functions in the eukaryotic cells, including Ca^2+^ homeostasis and post-translational modification of lipids and secreted proteins.^5,6^ It has been evidenced that some stressful insults, such as inflammation, hypoxia, and amino acid deficiency, may induce the accumulation of unfolded/misfolded proteins in the ER, leading to the activation of an adaptive response, called unfolded protein response (UPR).^5,6^ The primary goal of the UPR is to maintain the homeostasis and survival of cells. However, if the cells cannot cope with the stress, the UPR may switch into the programmed cell death.^7^ The UPR signaling is emanated from three ER transmembrane protein sensor(s), including PKR-like ER kinase (PERK), activated transcription factor 6 alpha (ATF6α) and inositol-requiring enzyme 1 alpha (IRE1α).^6,8,9^ In resting cells, the PERK, ATF6, and IRE1α (hereafter called IRE1) are associated with GRP78/Bip protein, while under the ER stress condition, GRP78 is dissociated from these UPR arms, resulting in the activation of UPR signaling.^7-9^ IRE1 is a unique enzyme with both kinase and RNase activities, which controls survival or cell death during ER stress.^10^ Under the ER stress condition, IRE1 is oligomerized and then its kinase domain is activated, leading to the activation of its RNase domain. The ribonuclease activity of IRE1 is responsible for the specific splicing of X-binding protein 1 (XBP1) mRNA and the regulated IRE1-dependent decay (RIDD).^11^ The spliced XBP1 (XBP1s) acts as a transcription factor and upregulates the genes related to ER folding capacity, membrane biogenesis, and ER quality control. In parallel, the RIDD targets a subset of mRNAs/miRNAs to decrease protein-folding demand or to induce cell death, depending on the tissue and stress types.^12^

It has been evidenced that dysregulation of the UPR pathways is associated with the pathogenesis and progression of RA.^13,14^ Most pathological hallmarks of RA, including hypoxia, low glucose, and excessive activation of immune responses,^15^ can exert a burden on the ER that may induce ER stress.^13,14^ This chronic ER stress can increase the rate of proliferation of synoviocytes, and the production of pro-inflammatory cytokines and autoantibodies in RA.^13-16^ The up-regulation of ER stress markers, including GRP78, IRE1, XBP1s, ATF6, and eIF2a-P, have been reported in macrophages and fibroblast-like synoviocytes (FLS) of RA patients.^16,17^ However, the functional importance of ER stress in RA needs more investigations.

Here, we report the increased RNase activity of the IRE1 in PBMCs from RA patients compared with healthy individuals. We further show that transcriptional changes at the downstream targets of IRE1 (especially miR-96 and XBP1s) may offer a new opportunity to improve the current diagnostic markers and therapeutic options in RA.

## Material and Methods

### Study design and patient’s selection

The case-control study involved 52 Iranian subjects, who were divided into two groups including patients and healthy controls (n=26). Patients with RA, defined by ACR/EULAR criteria, were selected during five months from March to July 2017 who referred to Imam Hossein hospital (Tehran, Iran) and diagnosed with active RA. A questionnaire was filled out by the volunteers to gather the demographic and other information related to this study. Healthy volunteers whose age and sex were matched with the patients group and had no previous report on cancer or any other chronic inflammatory disease were included. The clinical data of RA patients and healthy controls were summarized in Table 1.

**Table 1:** Clinical characteristics of rheumatoid arthritis patients (RA) and healthy controls

	**RA patients**	**Healthy controls**
Gender % (female/male)	89.28/10.72	81.25/18.75
Age mean (range) in years	52.6 (36-75)	50.6 (35-74)
Mean disease duration (years), range	6.2, 1-20	-
Smoking (%)	7.6	-
Active / sedentary work (%)	26.9/73.1	3.84/96.15
Vitamin D consumption (%)	42.30	12.5

### PBMC isolation and real-time PCR

A volume of 5 ml blood from all patients and controls was collected into EDTA tubes under aseptic condition. Blood samples were immediately transferred to the laboratory and PBMCs were isolated by a density gradient centrifugation method using Ficoll-Hypaque (Inno-train, Germany) according to the manufacturer’s protocol. Total RNA was extracted by RNA X plus (Sina Clone Co., Tehran, Iran) and the poly (A)-based mRNA/miRNA cDNA synthesis kit (Bon Yakhteh, Tehran, Iran) was used for cDNA synthesis. The expression of mRNA/miRNAs was measured by real-time PCR system (Illumina) using SYBR Premix, BON qPCR master mix and individual-specific primers that obtained from Bon Yakhteh Company (Tehran, Iran). Real-time PCR was performed according to the following thermal conditions: 95°C for 2 minutes, 40 cycles of 95°C for 5 seconds and 60°C for 30 seconds. The relative expression of transcript levels of each individual was calculated according to 2^-R∆∆CT^ and analyzed by Rest 2009 software. The expression levels of mRNAs and miRNAs were normalized to β-actin and snord, respectively.

### Reverse transcriptase (RT)-PCR

The specific primers to amplify the spliced and unspliced form of XBP1 mRNA were: forward 5’-AGCAAGGGGAATGAAGTGAG-3’ and reverse 5’-TGGGGAAGGGCATTTGAAGA-3’. The PCR condition was one cycle of denaturation (95°C for 5 minutes), 38 cycles of amplification (95°C for 25 seconds, 65 °C for 30 seconds, and 72°C for 30 seconds) and a final cycle of extension (72°C for 5 minutes). PCR products were electrophoresed on 2.5% agarose gel.

### Assessment of diagnostic value of ER stress

Receiver-operating characteristic (ROC) curve analysis was performed to evaluate the diagnostic value for IRE1 and miR-17, -34a, -96 and -125b. The 95% confidence interval (95% IC) of the area under the curve (AUC) were analyzed by ΔCt of each reaction using GenEx software version 6.

### Statistical analysis

All results were represented as mean ± SD from three independent experiments that performed in duplicates or triplicates. Normal distribution of data was determined by the Kolmogorov-Smirnov test using SPSS (Statistical Program for Social Science) software version 19. The Student’s *t* test was used to compare gene expressions between RA patients and healthy controls. The correlation coefficient was determined by the Spearman’s/Pearson’s correlation statics using GenEx software.

## Results and Discussion

**Figure 1:**
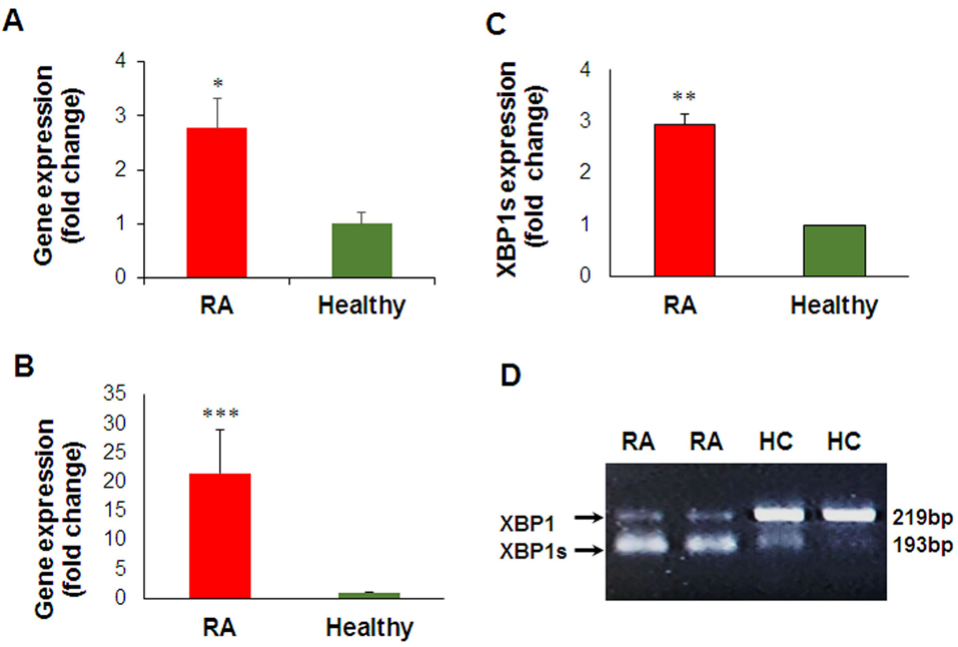
The expression of ER stress genes in RA patients in comparison to healthy ones. The gene expression levels *GRP78* (A), *IRE1* (B) and *XBP1s* (C) were evaluated by real-time PCR. The results are presented as mean ± SD.* *P* < 0.05, ** *P* < 0.01 and *** *P* < 0.001. D) The RT-PCR results of unspliced and spliced XBP1 (XBP1s) in PBMCs from two RA patients and healthy controls (HC). The gel electrophoresis image represents RT-PCR results obtained from isolated RNAs of each sample.

To investigate the involvement of ER stress in RA, we initially evaluated the transcriptional activation of two key UPR markers *ERN1* (*IRE1α)* and *HSP5A (GRP78/BiP*) in PBMC from RA patients (Figure 1). We focused on PBMCs because these samples are considered as a low-cost and non-invasive method for finding the new prognostic/therapeutic biomarkers.^18-20^ A significant increase in the expression levels of both *GRP78* (2.78-fold, *P* < 0.05) and *IRE1* (17.26-fold, *P* < 0.001) were observed in PBMCs from RA patients compared with healthy controls (Figure 1A and 1B). To determine the functional activation of IRE1/XBP1 axis of the UPR, we evaluated the splicing of *XBP1* in RA samples (Figure 1C). The real-time PCR results showed that *XBP1s* levels raised up to 3-fold in PBMCs from RA patients compared with healthy controls (Figure 1C). The semi-quantitative RT-PCR results also confirmed that the spliced form of XBP1 is observed in RA patients, while unspliced XBP1 is the dominant form of mRNA in healthy controls (Figure 1D). These results are in agreement with the previous reports, suggesting the upregulation of XBP1s in PBMC and FLS of RA patients.^16,17,21^ Recently, Kabala et al showed that the elevated level of XBP1s in RA was contributed to the apoptotic-resistant phenotype of FLS.^22^ It has been also reported that the IRE1/XBP1s axis of the UPR can contribute in the pathogenesis of RA by an increase at the level of autoantibodies produced against secreted GRP78.^23^ In this condition, the ER chaperone GRP78 acts as an autoantigen^24^ and contributes in the synoviocyte proliferation and angiogenesis, two hallmarks of RA pathogenesis.^16^ In addition, the upregulation of the GRP78 can selectively trigger the activation of T- and B-cells in RA.^13,25^ However, Savic et al demonstrated that the IRE1-mediated XBP1 splicing was unconventionally activated by toll-like receptor 2/4 (TLR 2/4) signaling without inducing the classic ER stress pathways.^21^ The authors also reported that the expression of other ER stress genes, including *SYNV1, GRP78* and *IRE1,* were downregulated in PBMCs of active RA patients compared with healthy groups.^21^ Yoo et al showed the high expression levels of the UPR mediators *CHOP*,*GRP78, IRE1*, and *ATF6* in the lining layer and/or sublining leukocytes of RA synovium.^16^ The cause of these discrepancies may be explained by difference in the types of samples and treatment protocols, as well as the context-dependency nature of this disease.

**Figure 2:**
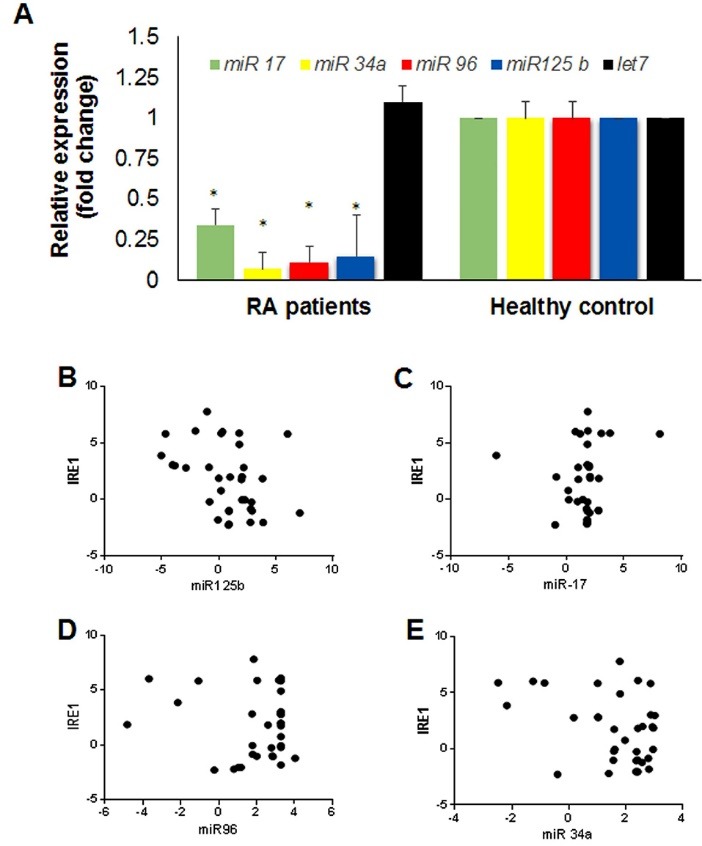
The expression level of downstream miRNAs targeted by IRE1 in RA patients. A) The expression levels of miR-17, miR-34a, miR-96, miR-125 and an irrelevant miR (let 7) was evaluated by quantitative real-time PCR in patients with RA in comparison to healthy ones. The results are presented as mean ± SD. Stars indicate P < 0.05. B-D) Correlation between IRE1 and miR-17, miR-34a, miR-96, miR-125b was performed by Spearman/Pearson test by GenEx software.

To further investigate the role of IRE1 in RA, we also studied the IRE1/RIDD pathway. The role of RIDD activity of the UPR is mostly unknown in RA.^14,26^ It has been reported that the RNase activity of IRE1 (both the XBP1 slicing and RIDD) may cause the release of pro-inflammatory cytokines, thereby exacerbate the pathogenesis of RA.^23^ Recent findings suggest that miRNAs are tightly regulated at multiple levels, ranging from their transcription to their decay by RNase enzymes.^23,27,28^ In this line, Upton et al reported that miR-17, miR-34a, miR-96 and miR-125b are degraded by RIDD activity of IRE1, leading to apoptosis induction via the upregulation of caspase-2 mRNA levels.^28^ Therefore, we studied the expression patterns of above-mentioned RIDD targets in RA patients. Our results showed that in contrast with IRE1 overexpression, the levels of *miR-17, miR-34a, miR-96* and* miR-125b* were significantly decreased in PBMCs of RA patients compared with healthy controls (Figure 2A). The expression level of let-7 miRNA was not significantly changed in this condition (Figure 2A). Quantitatively speaking, the average relative expression levels of *miR-17, -34a, -96* and* -125b* decreased up to 0.34-, 0.07-, 0.11-, and 0.15-fold, respectively (Figure 2A). The Pearson/Spearman correlation test revealed a negative correlation between IRE1 and its target miRNAs (Figure 2B-E), confirming the upstream activation of RIDD as an ER stress hallmark in PBMCs of RA patients. Very recently, Kabala et al showed that RIDD activity of IRE1 may modulate inflammatory responses via degrading anti-cytokine miRNAs in RA.^22^ Our results uncover that a wider range of miRNAs, such as anti-caspase-2 miRNAs, can be also regulated by IRE1/RIDD pathway in RA. The possible role of these miRNAs in the pathogenesis of RA needs more investigations. Regardless of the mechanism of action, the RNase activity of IRE1 may be a therapeutic target in RA.^4^

**Figure 3:**
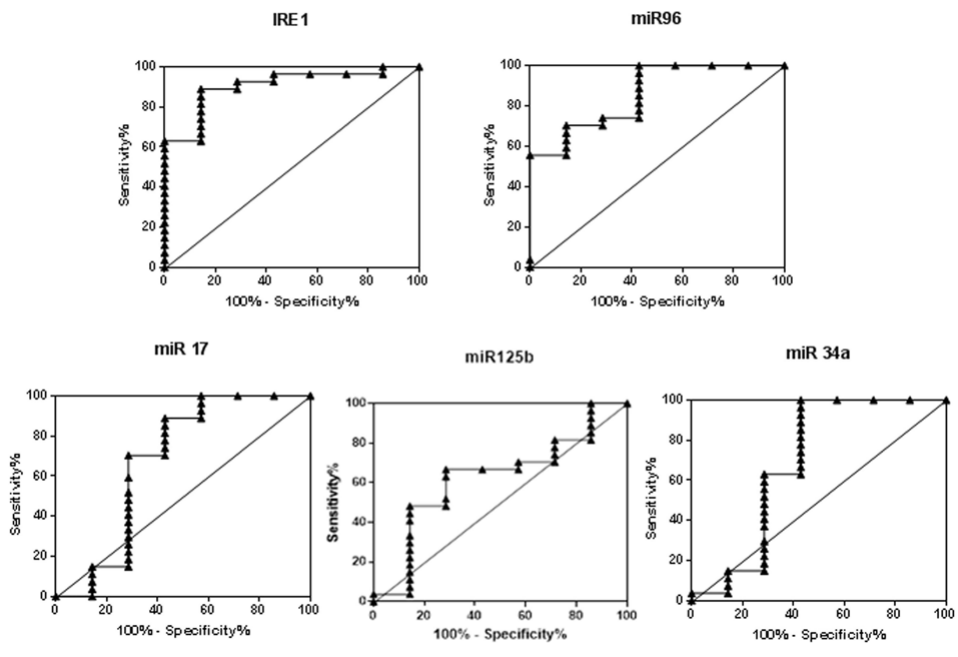
ROC curve of IRE1 and its targeted miRs in discrimination between RA patients and healthy controls. The AUC values for miR-17, miR-34a, miR-96, and miR-125b are 0.9048, 0.6772, 0.6878, 0.8571 and 0.6243, respectively.

Recent findings highlight the potential of miRNAs as a diagnostic biomarker in RA.^18-20^ Thus, we performed a ROC curve analysis to find if IRE1 and its downstream miRNAs have biomarker values in RA (Figure 3). The results (Figure 3) demonstrated that IRE1 and miR-96 may have moderate diagnostic values for the diagnosis of RA. However, larger sample sizes and more experiments are required to support these findings. In conclusion, the RNase activity of IRE1 may offer a new opportunity to improve the current therapeutic and/or diagnostic markers in RA patients.

## Ethical Issues

This study was carried out according to Declaration of Helsinki and under Tehran University of Medical Sciences research ethics (code: IR.TUMS.REC.1395.2320).

## Conflict of Interest

Authors declare no conflict of interest in this study.

## Acknowledgments

This work has been supported financially by the Iran National Science Foundation (INSF) project no. 94807020. MR acknowledges financial supports from Cancer Biology Research Center, Tehran University of Medical Sciences. AM is supported by grant (No: 980301-I-728) from National Institute of Genetics Engineering and Biotechnology (NIGEB).
